# Defense Responses in Grapevine (cv. Mourvèdre) after Inoculation with the Botryosphaeria Dieback Pathogens *Neofusicoccum parvum* and *Diplodia seriata* and Their Relationship with Flowering

**DOI:** 10.3390/ijms18020393

**Published:** 2017-02-13

**Authors:** Alessandro Spagnolo, Vincenzo Mondello, Philippe Larignon, Sandra Villaume, Fanja Rabenoelina, Christophe Clément, Florence Fontaine

**Affiliations:** 1 Structure Fédérative de Recherche (SFR) Condorcet-FR CNRS 3417, Université de Reims Champagne-Ardenne, Unité de Recherche Vignes et Vins de Champagne EA 4707, Laboratoire Stress, Défenses et Reproduction des Plantes, BP 1039, (Cedex 2), 51687 Reims, France; alessandro.spagnolo@univ-reims.fr (A.S.); vincenzo.mondello@univ-reims.fr (V.M.); sandra.villaume@univ-reims.fr (S.V.); clarisse.rabenoelina@univ-reims.fr (F.R.); christophe.clement@univ-reims.fr (C.C.); 2 Institut Français de la Vigne et du Vin Pôle Rhône-Méditerranée, 7 Avenue Cazeaux, 30230 Rodilhan, France; philippe.larignon@vignevin.com

**Keywords:** Botryosphaeria dieback, *Neofusicoccum parvum*, *Diplodia seriata*, defense responses, flowering

## Abstract

As a result of the increasing economic impact of grapevine trunk diseases on viticulture worldwide, efficient and viable control strategies are urgently needed. However, understanding both plant-pathogen interactions and plant physiological changes related to these diseases is fundamental to such an achievement. In this study, we analyzed the effect of inoculation with the Botryosphaeria dieback fungal agents, *Neofusicoccum parvum* and *Diplodia seriata*, with and without inflorescence removal at the onset of G stage (separated clusters), I stage (flowering) and M stage (veraison). A measure of lesion size and real-time reverse-transcription polymerase chain reaction-based analysis were carried out. The results clearly show the importance of inflorescences in the development of lesions associated with Botryosphaeria dieback pathogens inoculated on green stems of adult vines, especially at the onset of flowering. At flowering, the biggest necroses were observed with the inflorescences present, as well as an activation of the studied defense responses. Thus, an ineffective response to the pathogen could be consistent with a possible metabolic reprogramming linked to the host phenophase.

## 1. Introduction

Grapevine trunk diseases (GTDs) commonly represent the most destructive diseases of grapevine worldwide [1,2]. Symptoms consist of wood discolorations and sometimes specific foliar symptoms associated with a general vigor decline, which finally leads to premature plant death. Causal agents are a number of taxonomically unrelated fungi, which colonize the grapevine wood basically through pruning wounds [1,3]. Botryosphaeria dieback [4] caused by species in the Botryosphaeriaceae [5] is probably the most widespread GTD in the world’s grape growing regions [6]. As reported earlier [6], additional ways of penetration for the Botryosphaeriaceae could also be represented by wounds occurring during the vegetative growth period (e.g., removal of lateral shoots and desuckering, or climatic events, such as strong wind and hail). Indeed, the airborne inoculum is present particularly during rainfall [7,8] with peak release during the vegetative growth period [9,10].

After the banning of sodium arsenite [1,2], which was the most highly effective compound against GTDs to date, efficient control strategies for GTDs are presently more urgent, due to the increasing economic impact of GTDs on viticulture [2]. Furthermore, the research results of the last twenty years seem to highlight the role of plant-pathogen interactions and of plant physiological changes on the GTD disease development and symptoms expression.

One of the most relevant factors in plant physiology changes is carbohydrate metabolism. Carbohydrates are not only the fundamental constituent of structural and storing-energy polymers as cellulose and starch, but also the substrate and the energy sources in almost all bio-chemical pathways of plant cells. In cultivated plants and especially in grapevines, nonstructural carbohydrates (i.e., starch and sugars) are able to move inside the plant according to the phenological stage and play a critical role in plant metabolism, especially for the reproductive phase [11]. Grape berry development and ripening represent a significant event for grapevine physiology and for carbohydrate metabolism. The ecological importance of this phase was stressed also by studies that demonstrated how carbohydrates are mobilized toward the reproductive organs in diseased grapevines, to compensate the losses determined by the biotic stresses [12].

Currently, studies focused on plant defense responses have indicated significant impacts on plant physiology and on primary metabolism. The activation of these mechanisms as a reaction to biotic stresses could represent, in fact, a great effort in terms of energetic needs, leading to growth and fitness reduction in plants [13]. Among plant defense mechanisms, the latent ones, induced by pathogenetic processes, lead to changes in cell metabolism, mainly in host plant gene expressions, as observed in *Arabidopsis* [14].

A recent study focused on physiological changes occurring in GTD-affected grapevines has highlighted that defense responses are activated in infected plants (antioxidant system, phenylpropanoid pathway, PR-proteins, etc.), but these are probably not enough to avoid the disease development [15]. In a preliminary study aimed at identifying the sensitivity of grapevine to the Botryosphaeria dieback agents *Neofusicoccum parvum* and *Diplodia seriata* infection during the growth season [6], the flowering phase was designated as being the period of highest weakness. The authors hypothesized that this condition could be due to a reduced reactiveness of grapevine as a consequence of a metabolic activity basically oriented towards the developing inflorescences during such a phenophase.

According to this hypothesis, the goal of this study was to gain further insights into the physiological changes occurring in green stems of adult vines cv. Mourvèdre artificially infected with Botryosphaeria dieback pathogens. Notably, we analyzed the effect of inflorescences removal at the F stage (visible clusters) on the evolution of artificial inoculations with the agents *N. parvum* and *D. seriata*, on the onset of G stage (separated clusters), of I stage (flowering) and at the M stage (veraison). The measure of lesion size and real-time reverse-transcription polymerase chain reaction (RT-qPCR)-based analysis were carried out.

## 2. Results

### 2.1. Pathogenicity Tests

Both pathogens, *N. parvum* and *D. seriata*, were always re-isolated from the edge of the lesions associated with their artificial inoculation; thus, Koch’s postulates were accomplished in both experiments. No fungi were isolated from the lesion of control stems, indicating its development only as a consequence of the artificial wound.

#### 2.1.1. Artificial Inoculation with *N. parvum*

**Figure 1:**
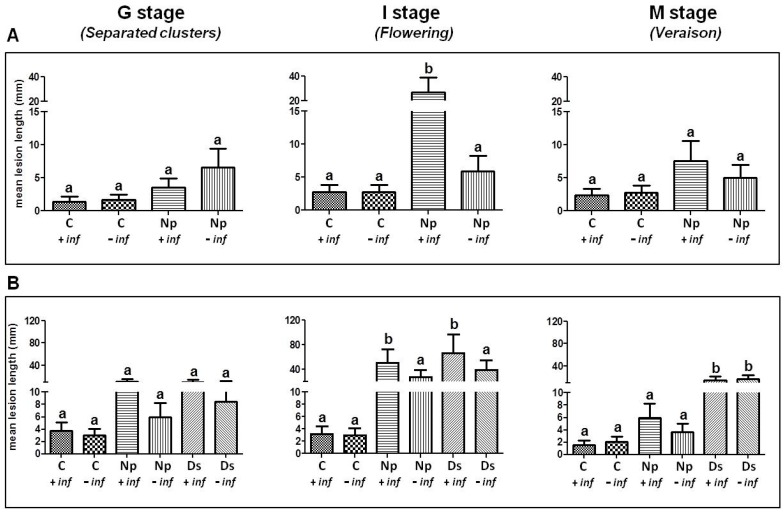
Mean lesion lengths ± SE on green stems after artificial inoculation at the onset of the G stage, flowering and veraison with: (**A**) *N. parvum* (Np) and (**B**) *N. parvum* (Np) or *D. seriata* (Ds) the in presence (+inf) or absence (−inf) of inflorescences; control stems (C + inf, C − inf) were wounded and inoculated with sterile malt agar. Differences among the means were evaluated by Dunn’s multiple comparison test; after that the null hypothesis (equal means) was rejected in the Kruskal–Wallis test, assuming a significance of *p* ≤ 0.05. The same letter above columns indicates no statistically significant differences for *p* ≤ 0.05.

*N. parvum* (Np) was inoculated on green stems of adult standing vines from which inflorescences had or not previously been removed. In all three stages, lesion lengths in control stems (C + inflorescence (inf), C − inf) were lower than those in the inoculated ones. These differences reached a statistical significance at stage I only in the inoculated stems with inflorescences. Results show that the longest lesions developed on green stems inoculated at the onset of I stage (flowering) and M stage (veraison) (Figure 1A). Indeed, mean lesion lengths were: 11.5 ± 2.6, 21.8 ± 3 and 18.7 ± 6.4 mm for G, I and M stages, respectively. Differences between inoculated stems with (Np + inf) or without inflorescences (Np − inf) were substantial at I stage (flowering) where lesions measured 27.2 ± 11.8 and 5.9 ± 2.4 mm, respectively. While not statistically significant differences between Np − inf and Np − inf were also recorded for G and M stages, while the presence/absence of inflorescences had likely no influence in both control stems, where lesions were due only to the effect of the tissues oxidation on the wounded area and were not determined by other biological agents.

#### 2.1.2. Artificial Inoculation with *D. seriata* and *N. parvum*

According to the wide range of pathogens associated with Botryosphaeria dieback and to their ascertained different pathogenicity towards *V. vinifera*, the same experiment was repeated using *N. parvum* (Np) and *D. seriata* (Ds) in artificial inoculations (Figure 1B). Similar to the previous trial, the lower lesion length was recorded in control stems, with statistically-significant differences at I stage compared to the inoculated stems carrying inflorescences, irrespective of the inoculated pathogen. Again, the longest lesion sizes were noted at the I stage (flowering) on the inoculated stems carrying the inflorescences (Np + inf and Ds + inf). In detail, lesion lengths were 51.3 ± 22.0 and 66.6 ± 30.2 mm, for *N. parvum* and *D. seriata*, respectively. The corresponding inoculated treatment without inflorescences showed lower values, being 27.9 ± 11.2 mm for *N. parvum* and 39.8 ± 18.8 mm for *D. seriata*. The differences in lesion length between the “+ inf” and “− inf” thesis were statistically significant.

The longest lesion lengths on stems inoculated in the presence of inflorescences were also recorded at G stage for both *N. parvum* and *D. seriata* and for *N. parvum* only at the M stage (veraison). No statistically-significant differences were recorded in these cases. Contrary to what occurred at the G stage and flowering, *D. seriata* caused longer lesions (15 ± 6.2 and 17.2 ± 7.3 mm) than *N. parvum* (6.0 ± 2.3 and 3.7 ± 1.3 mm) at veraison, irrespective of the presence or absence of inflorescences.

### 2.2. Transcript Analysis

Expression analysis of a selected set of genes was performed for samples from all of the experiments conducted in this study. The panel of genes, which were chosen based on results of previously published studies [6,16,17,18,19], included genes encoding components of the phenylpropanoid pathway, PR and other plant defense proteins, proteins involved in the detoxification processes, as well as in primary metabolism or water stress.

#### 2.2.1. Inoculations with *N. parvum*

**Figure 2:**
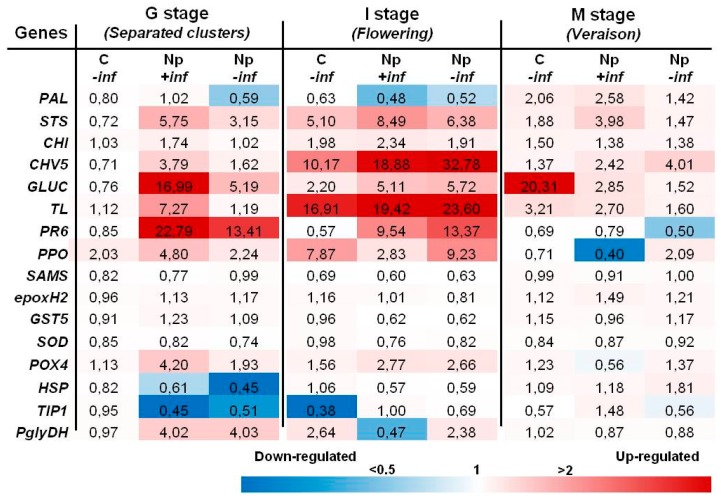
Expression levels of the selected 16 genes recorded by RT-qPCR in *N. parvum* artificially-inoculated stems at different phenological stages. Values (the mean of three technical replicates) represent the expression levels (ΔΔ*C*_t_) of reported conditions relatively to the control (C + inf). Expression of a given gene was considered up- or down-regulated when the value of relative expression was >2-fold or <0.5-fold compared to the control, respectively. Due to the similarity to control values, the last eight genes were not considered in the following trials.

Most of changes consisted of the upregulation in stems inoculated at the onset of G stage (separated clusters) and I stage (Figure 2). At the G stage, eight genes, including *STS*, *CHI*, *PR* genes, *PPO*, *POX4* and *PglyDH*, were upregulated in Np-inoculated stems with inflorescences (Np + inf). The highest values were recorded for the *PR* genes *GLUC* and *PR6*, which rose up to about 17- and 23-fold as compared to the control, respectively. Five of them were likewise upregulated, although to a lesser extent, in Np-inoculated stems without inflorescences (Np − inf), where *PR6* expression was 13-fold. Most of these genes were also induced at flowering where the highest levels of induction were noted for *CHV5* (about 32-fold) and *TL* (about 24-fold) in *N. parvum* stems without inflorescences (Np − inf). Upregulation of the phenylpropanoid pathway and *PR* genes was still detected at the M stage (veraison), but with lower induction levels with regard to the previous G and I stages. In fact, the sole expression level higher than 10-fold at veraison was detected for *GLUC* without inflorescences (C − inf).

#### 2.2.2. Inoculations with *D. seriata* and *N. parvum*

**Figure 3:**
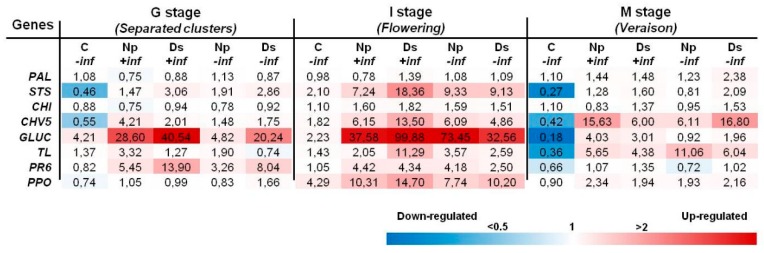
Expression levels of the considered eight genes recorded by RT-qPCR in *N. parvum* and *D. seriata* artificially-inoculated stems at different phenological stages. Values (the mean of three technical replicates) represent the expression levels (ΔΔ*C*_t_) of reported conditions relative to the control (C + inf). The expression of a given gene was considered up- or down-regulated when the value of relative expression was >2-fold or <0.5-fold compared to the control, respectively.

Based on results of the first inoculation trial, only eight genes (*PAL*, *STS*, *CHI*, *CHV5*, *GLU*, *TL*, *PR6* and *PPO*) were retained for transcript analysis of the second one. Results indicate that changes mostly consisted in an upregulation, which involved the highest number of genes at the I stage (flowering). Some different trends of induction level between *N. parvum*- and *D. seriata*-inoculated stems were recorded at all stages. The induction in stems with *D. seriata* was the highest in the presence of inflorescences, especially at the G and I stages (Figure 3). Among the genes upregulated at the G stage (*STS*, *CHV5*, *GLUC*, *TL* and *PR6*), the *GLUC* showed the maximal levels of induction with 28.60-fold and 40.54-fold in Np + inf and Ds + inf, respectively. Moreover, *GLUC* was the only one to be upregulated in control stems (C − inf). The same genes were even more induced at the I stage (flowering) where the highest levels were detected for *GLUC*, which rose up to 99-fold and 73-fold for Ds + inf and Np − inf, respectively. *PPO* also was amongst those genes upregulated and the only one to be fairly induced in control stems at I stage (flowering). At the M stage (veraison), only three *PR* genes, namely *CHV5*, *GLUC* and *TL*, were upregulated in all inoculated stems, irrespective of inflorescence presence or absence, except for *GLUC* in Np − inf. No gene upregulation was recorded at this stage in control stems where, on the contrary, four genes (*STS*, *CHV5*, *GLUC*, *TL*) were downregulated.

## 3. Discussion

### 3.1. Pathogenicity Tests

Similarly to our previous trials [6], flowering was the stage at which vines were more susceptible to the two botryosphaeriaceous fungi. In fact, the highest lesions were recorded during this phenophase. Furthermore, the results obtained in the two inoculation trials showed also that *N. parvum* and *D. seriata* were able to produce higher lesion length in green stems with inflorescences than in those without inflorescences, with few exceptions. These results confirm the hypothesis that the flowering stage is the period of highest sensitivity to Botryosphaeria dieback agents, as a consequence of the high metabolic activity oriented towards the inflorescences’ development.

This could effectively mean that the particular metabolism imposed by the developing inflorescences [20] has an impact on the defense efficiency of grapevine in such a period [21]. Moreover, increased carbohydrate availability in green stems during the advanced phase of flowering [20,22] could have further enabled wood-colonizing pathogen infection.

Independently of the presence/absence of inflorescences, mean lesion sizes associated with *N. parvum* and *D. seriata* observed in the second inoculation trial were longer than those registered in the first experiment or in a previous related study [6]. This could be due to an increased virulence of the pathogens, which in turn could depend on different climatic conditions occurred during the vegetative seasons [23,24]. On the other hand, the same climatic variations could also have determined a lower responsiveness of the inoculated plants, since it was attested that cultural practices in vineyard and climatic factors could influence host plant metabolism, for instance determining different carbohydrate accumulation degrees in reserve organs and/or higher mobility during the vegetative and reproductive phases [25,26].

### 3.2. Transcript Analyses

Similar to pathogenicity test results, flowering (I stage) was the phenological phase in which the highest gene induction values were recorded in both inoculation trials, regardless of the presence or absence of inflorescences. Especially during flowering, the artificial inoculation with *N. parvum* and *D. seriata* induced the upregulation of several plant defense-related genes. Interestingly, this upregulation at flowering was also observed in non-inoculated control stems without inflorescences, even if this was to a lesser extent. Gene induction in the two experiments basically concerned phenylpropanoid pathway (*STS*, *CHI*) and *PR* genes (*CHV5*, *GLUC*, *TL*, *PR6*).

Transcriptomic results of the first inoculation trial are consistent with those of the related pathogenicity tests. Defense responses at the I stage (flowering) were generally stronger in stems without inflorescences inoculated with *N. parvum* if compared to the corresponding inoculated ones carrying inflorescences. These differences could have determined the lower lesions size recorded in the –inf inoculated stems. Beyond the above-mentioned genes, upregulation of *POX4*, especially noted at the G stage and to a lesser extent at the I stage, may be indicative of a cell-wall reinforcement activity [19], as well as of a resveratrol oligomer synthesis [27] resulting from the pathogen’s activity. Similarly to *POX4*, induction of *PglyDH* indicates a possible intensification of primary metabolism during these two phenophases as a reaction to infection, especially at the G stage. At the I stage, its upregulation was induced by the inflorescences removal rather than the pathogen’s presence. This supports both the evidence that cost-intensive carbohydrate metabolism [13,28] plays an important role in plant defense response activation [28,29] and that inflorescence presence could hamper this response during flowering, if infections occur.

At the I stage (flowering) of the second trial, the influence of inflorescence removal in *N. parvum*-inoculated stems was less marked than in the first one, especially when compared to *D. seriata*-inoculated stems. Different from the first inoculation trial results, transcript analysis appeared to not corroborate those of pathogenicity tests. The host plant defense response, which was apparently stronger in the presence of inflorescences, was not accompanied by reduced lesion lengths. The transcript results of the second inoculation trials have also shown a shift in the genes’ induction, if compared to the first ones, especially at flowering. In particular, according to their upregulation values, the most induced genes were *CHV5*, *TL*, *PR6* and *GLUC* in the first trial and *GLUC*, *STS*, *CHV5* and *PR6* in the second one. These differences at the transcriptomic level could probably play a role in plant defense efficiency in controlling the pathogen aggressiveness. However, as for lesion lengths, this could depend on climatic variations occurring during the two vegetative seasons [23,24], which would have influenced both plant responsiveness and fungal aggressiveness.

The expression of some of these genes was recently analyzed by Reis et al. [30] after artificial infection with *N. parvum* and *D. seriata* strains on green stems of greenhouse-trained one-year-old vines cv. Tempranillo. In that study, induction of *STS* in *N. parvum*-infected stems corresponded to a significant accumulation of trans-resveratrol. Basing on that observation, already hypothesized by Spagnolo et al. [31], the expression trends of *CHI* and *STS* could indicate that the phenylpropanoid pathway, which leads to the synthesis of stilbenes, was favored over the flavonoid pathway. Phenylpropanoids have a role in plant defense, and their functions range from preformed or inducible physical and chemical barriers against infection to molecules involved in local and systemic signaling for defense gene induction [32]. Among phenolic compounds, stilbenes are thought to play a role in limiting the development of fungi in grapevine wood [13,33,34]. Apart from its classical antimicrobial activity, the stilbene resveratrol has also been observed acting as a signaling molecule activating defense-related responses in *Vitis* cells [35]. Upregulation of *PR* genes has previously been reported in naturally [17,19,36,37] and artificially [38] GTD-infected plants, as well as in grapevine cells in the presence of extracellular compounds produced by Botryosphaeria dieback pathogens [39].

In any case, the results in this study seem to confirm, even if only at the transcript level, the trends of protein abundance reported by Spagnolo et al. [6], where a general protein over accumulation including defense-related proteins was registered at the G stage while a very small amount of protein species was differentially expressed at flowering. While ignoring the causes of such discrepancy, already observed by Spagnolo et al. [31], this could be the result of the relatively shorter half-life of mRNAs compared to proteins or of a specific condition related to the flowering, which could manifest itself following post-transcriptional regulation [40].

The putative over accumulation of β-1,3-glucanase also in non-wounded stems at flowering emphasizes multiple roles for proteins belonging to this group. These enzymes, copious in plants, are involved in several processes during cell division, as the passage of materials through plasmodesmata, and allow plants to tolerate abiotic stresses. β-1,3-glucanase also defends plants against fungal pathogens being able to degrade fungal cell walls either alone or in association with other antifungal proteins (e.g., chitinase isozymes) [41].

## 4. Materials and Methods

### 4.1. Plant Material, Fungal Strains and Artificial Inoculations

The experimental site was a vineyard of cultivar Mourvèdre/3309 planted in 1997, located at Rodilhan (Nîmes, France) and owned by the Lycée agricole Marie-Durand of Rodilhan. The study consisted of two experiments which were performed in two different vegetative seasons between 2013 and 2014. All of the experiments provided artificial inoculation with fungal species on green stems at the onset of the phenological stages G (separated clusters), I (flowering) and M (veraison) after manual removal or not of inflorescences at the F stage (visible clusters), according to the Baggiolini phenological scale [42]. Controls consisted of stems wounded and inoculated with sterile malt agar with or without inflorescences (C + inf, C − inf). The following fungal species were used in the study: the Botryosphaeria dieback agents (i) *Neofusicoccum parvum* (strain Np SV isolated from symptomatic vines in Aix-en-Provence, France); and (ii) *Diplodia seriata* (strain Bo 98.1 isolated from symptomatic vines in Perpignan, France).

Stems were longitudinally wounded with a sterile scalpel at the level of the third internode. The wounds (8 mm length, 1 mm deep) were inoculated by putting a 5-mm diameter plug taken from the edge of a 5-day-old actively-growing fungal culture. Controls were inoculated with sterile media plugs. The inoculation sites were then covered with parafilm. Each condition was replicated 8 times (one repetition per plant) for a total of 96 vines for the first inoculation trial and 144 for the second one. In autumn, five replications of each condition from all phenological stages were utilized to perform observation on lesions development and re-isolation tests, as described by Larignon and Dubos [9]. Samples for RNA extraction (3 out of 8 repetitions) were collected 20 days after inoculation and consisted of the portion of the inoculated internode (the corresponding wounded internode, for controls). Immediately after collection, samples were frozen in the field with liquid nitrogen and subsequently stored at −80 °C. Before RNA and/or protein extraction, the amount of biological sample needed was ground to a fine powder in liquid nitrogen with a Mixer Mill MM 400 (Retsch, Haan, Germany).

### 4.2. Experimental Design

The two inoculations trials provided either the sole *N. parvum* or both *N. parvum* and *D. seriata* as pathogens for artificial inoculations in the second year (Table 1). Each pathogen was individually inoculated in the presence or absence of inflorescences. For each phenological stage, green stems wounded and inoculated with sterile malt agar (C2) were the control stems. All of the conditions were considered for transcript analysis.

### 4.3. Transcript Analysis

#### 4.3.1. RNA Extraction

PlantRNA Purification Reagent (Thermo Fischer Scientific Inc., Waltham, MA, USA) was used to extract total RNA from 2 × 50 mg of powdered green stem tissues and was DNase treated. The quality of RNA was checked by agarose gel electrophoresis, and the quantity was determined by measuring the absorbance at 260 nm.

#### 4.3.2. Real-Time RT-PCR Analysis of Gene Expression

Reverse transcription was performed on 150 ng of total RNA using the Verso cDNA synthesis kit (Thermo Fischer Scientific Inc.). Real-time PCR was performed with Absolute Blue QPCR SYBR Green (Thermo Fischer Scientific Inc.) using a CFX96 thermocycler system (Bio-Rad, Hercules, CA, USA). The thermal profile was: 15 s at 95 °C (denaturation) and 1 min at 60 °C (annealing/extension) for 40 cycles. Melting curve assays were performed from 65–95 °C at 0.5 °C·s^−1^. Melting peaks were visualized to check the specificity of each amplification. Results are expressed as the values of relative expression (ΔΔ*C*_t_) and correspond to the mean from three independent experiments. The genes analyzed were considered significantly up- or down-regulated when changes in their expression were >2-fold or <0.5-fold, respectively. The specific primers for the 16 target genes are listed in Table 2.

## 5. Conclusions

The results of this study clearly show the host-plant gene induction determined by the two Botryosphaeria dieback pathogens *N. parvum* and *D. seriata* when infecting green stems of adult standing vines, confirming the activation of defense response toward these pathogens in grapevine. Furthermore, pathogenicity tests attested to the importance of inflorescences and of the grapevine phenological phase in the development of lesions associated with Botryosphaeria dieback, especially at the onset of flowering. As a matter of fact, lesions were shorter when pathogens were inoculated after inflorescence removal. Moreover, the general increase of gene induction at flowering suggests that defense responses following wounding or artificial inoculation with Botryosphaeria dieback pathogens are also activated, at least at the transcript level, and these likely are even stronger than at the G stage. On the other hand, the presence of inflorescences may constitute the reason for an ineffective response (longer lesions) as a consequence of a possible metabolism reprogramming linked to the particular phenophase. This could be represented by the reorientation of metabolism towards developing inflorescences, which can reduce energy availability (e.g., for protein synthesis) in neighbor tissues at the onset of the flowering and/or by the reserve replenishment in the same organs during the advanced phase of the phenological stage.

The differences in gene induction between the two vegetative seasons recorded in *N. parvum* inoculations and the similar ones recorded both for *N. parvum* and *D. seriata* within the same season seem to evidence the non-pathogen-specific nature of the observed defense responses in grapevine. As for other polygenic-based resistances, climate could represent a key-factor able to “modulate” the expression of single genes and, thus, the global effect of genes’ induction. This hypothesis agrees with the results of Spagnolo et al. [31] that highlighted a common pool of upregulated plant defense-related genes, beside other, differentially-induced genes, according to the cultivar growing under different climates. All together with the influence of climate on pathogens’ fitness and aggressiveness, this could explain the different length of lesion associated with pathogen inoculations.

Based on these findings, further studies should be addressed to determine those factors/mechanisms related to inflorescences that would influence the metabolism and responses to stress in green stems during flowering.

## Figures and Tables

**Table 1:** Conditions and sample codes.

Experiment	Condition	Sample Codes		
		G Stage (*Separated Clusters*)	I Stage (*Flowering*)	M Stage (*Veraison*)
**Inoculation Trial No. 1 and 2**	Control	C − inf/C + inf	C − inf/C + inf	C − inf/C + inf
	*N. parvum* strain Np SV	Np − inf/Np + inf	Np − inf/Np + inf	Np − inf/Np + inf
**Inoculation Trial No. 2**	*D. seriata* strain Bo98.1	Ds − inf/Ds + inf	Ds − inf/Ds + inf	Ds − inf/Ds + inf

Legend: C = Control; Np = *N. parvum*; Ds = *D. seriata*; + inf/− inf = with/without inflorescences.

**Table 2:** Primers of genes analyzed by real-time reverse-transcription polymerase chain reaction.

Function	Gene	Primer Sequences	GenBank or TC TIGR * Accession Number
*Housekeeping genes*	*EF1* (EF1-α elongation factor)	5′-GAACTGGGTGCTTGATAGGC-3′	GU585871
		5′-AACCAAAATATCCGGAGTAAAAGA-3′	
	*60SRP* (60S ribosomal protein L18)	5′-ATCTACCTCAAGCTCCTAGTC-3′	XM_002270599
		5′-CAATCTTGTCCTCCTTTCCT-3′	
*Phenylpropanoid metabolism*	*CHI* (Chalcone isomerase)	5′-GCAGAAGCCAAAGCCATTGA-3′	NM_001281104
		5′-GCCGATGATGGACTCCAGTAC-3′	
	*PAL* (Phenylalanine ammonia lyase)	5′-TCCTCCCGGAAAACAGCTG-3′	X75967
		5′-TCCTCCAAATGCCTCAAATCA-3′	
	*POX4* (Peroxidase-like 4)	5′-AACATCCCCCCTCCCACTT-3′	XM_002269882
		5′-TGCATCTCGCTTGGCCTATT-3′	
	*STS* (Stilbene synthase)	5′-AGGAAGCAGCATTGAAGGCTC-3′	FJ851185
		5′-TGCACCAGGCATTTCTACACC-3′	
*Defense protein*	*CHV5* (Chitinase class v)	5′-CTACAACTATGGCGCTGCTG-3′	AF532966
		5′-CCAAAACCATAATGCGGTCT-3′	
	*GLUC* (β-1,3 glucanase)	5′-TCAATGGCTGCAATGGTGC-3′	DQ267748
		5′-CGGTCGATGTTGCGAGATTTA-3′	
	*PPO* (Polyphenol oxidase)	5′-TGGTCTTGCTGATAAGCCTAGTGA-3′	XM_002727606
		5′-TCCACATCCGATCGACATTG-3′	
	*PR6* (Serine-protease inhibitor 6)	5′-AGGGAACAATCGTTACCCAAG-3′	AY156047
		5′-CCGATGGTAGGGACACTGAT-3′	
	*SAMS* (S-adenosylmethionine synthetase)	5′-CCTGAAATCAAAGTTCTCCTTCACA-3′	XM_002266322
		5′-CCGGGCCTGAAATCAAAGTT-3′	
	*TL* (Thaumatin-like)	5′-CCTAACACCTTAGCCGAATTCGC-3′	AF532965
		5′-GGCCATAGGCACATTAAATCCATC-3′	
*Detoxification and Stress tolerance*	*epoxH2* (Epoxide hydrolase 2)	5′-TCTGGATTCCGAACTGCATTG-3′	XM_002270484
		5′-ACCCATGATTAGCAGCATTGG-3′	
	*GST5* (Glutathione s-transferase 5)	5′-GCAGAAGCTGCCAGTGAAATT-3′	XM_002277883
		5′-GGCAAGCCATGAAAGTGACA-3′	
	*HSP* (alpha crystalline heat shock protein)	5′-TCGGTGGAGGATGACTTGCT-3′	XM_002272382
		5′-CGTGTGCTGTACGAGCTGAAG-3′	
	*SOD* (Superoxide dismutase)	5′-GTGGACCTAATGCAGTGATTGGA-3′	AF056622
		5′-TGCCAGTGGTAAGGCTAAGTTCA-3′	
*Primary metabolism*	*PglyDH* (Phosphoglycerate dehydrogenase)	5′-CGTCGAAGATGCTCAATGATGA-3′	XM_002285322
		5′-CCCCCACGAGCAACATTAATT-3′	
*Water stress*	*TIP1* (Tonoplast intrinsic protein)	5′-ATCACCAACCTCATTCATATGC-3′	AF271661
		5′-GTTGTTGTCTCAACCCATTTCC-3′	

* see http://www.jcvi.org/cms/research/projects/tdb/overview/.

## References

[1] Bertsch C., Ramirez-Suero M., Magnin-Robert M., Larignon P., Chong J., Abou-Mansour E., Spagnolo A., Clément C., Fontaine F. (2013). Grapevine trunk diseases: Complex and still poorly understood. Plant Pathol..

[2] Fontaine F., Gramaje D., Armengol J., Smart R., Nagy Z.A., Borgo M., Rego C., Corio-Costet M.-F. (2016). Grapevine Trunk Diseases. A Review.

[3] Di Marco S., Reggiori F., Baleani M., Benanchi M., Bossio D., Osti F., Mugnai L. (2014). New wound infections are really relevant for grapevine leaf stripe disease? The case of *Trichoderma* pruning wound protection. Phytopathol. Mediterr..

[4] Úrbez-Torres J.R. (2011). The status of Botryosphaeriaceae species infecting grapevines. Phytopathol. Mediterr..

[5] Chethana K.W.T., Li X.H., Zhang W., Hyde K.D., Yan J.Y. (2016). Trail of decryption of molecular research on Botryosphaeriaceae in woody plants. Phytopathol. Mediterr..

[6] Spagnolo A., Larignon P., Magnin-Robert M., Hovasse A., Cilindre C., van Dorsselaer A., Clément C., Schaeffer-Reiss C., Fontaine F. (2014). Flowering as the most highly sensitive period of grapevine (*Vitis vinifera* L. cv. Mourvèdre) to the Botryosphaeria dieback agents *Neofusicoccum parvum* and *Diplodia seriata* infection. Int. J. Mol. Sci..

[7] Van Niekerk J.M., Calitz F.J., Halleen F., Fourie P.H. (2010). Temporal spore dispersal patterns of grapevine trunk pathogens in South Africa. Eur. J. Plant Pathol..

[8] Úrbez-Torres J.R., Bruez E., Hurtado J., Gubler W.D. (2010). Effect of temperature on conidial germination of Botryosphaeriaceae species infecting grapevines. Plant Dis..

[9] Larignon P., Dubos B. (1997). Fungi associated with esca disease in grapevine. Eur. J. Plant Pathol..

[10] Kuntzmann P., Villaume S., Larignon P., Bertsch C. (2010). Esca, BDA and Eutypiosis: Foliar symptoms, trunk lesions and fungi observed in diseased vinestocks in two vineyards in Alsace. Vitis.

[11] Zufferey V., Murisier F., Vivin P., Belcher S., Lorenzini F., Spring J.L., Viret O. (2012). Carbohydrate reserves in grapevine (*Vitis vinifera* L. “Chasselas”) the influence of the leaf to fruit ratio. Vitis.

[12] Jermini M., Blaise P., Gessler C. (2010). Quantification of the influence of the downy mildew (*Plasmopara viticola*) epidemics on the compensatory capacities of Vitis vinifera “Merlot” to limit the qualitative yield damage. Vitis.

[13] Bolton M.D. (2009). Primary metabolism and plant defense—Fuel for the fire. Mol. Plant Microbe Interact..

[14] Katagiri F. (2004). A global view of defense gene expression regulation—A highly interconnected signaling network. Curr. Opin. Plant Biol..

[15] Fontaine F., Pinto C., Vallet J., Clément C., Gomes A.C., Spagnolo A. (2016). The effects of grapevine trunk diseases (GTDs) on vine physiology. Eur. J. Plant Pathol..

[16] Magnin-Robert M., Spagnolo A., Boulanger A., Joyeux C., Clément C., Abou-Mansour E., Fontaine F. (2016). Changes in plant metabolism and accumulation of fungal metabolites in response to esca proper and apoplexy expression in the whole grapevine. Phytopathology.

[17] Magnin-Robert M., Letousey P., Spagnolo A., Rabenoelina F., Jacquens L., Mercier L., Clément C., Fontaine F. (2011). Leaf strip form of esca induces alteration of photosynthesis and defence reactions in presymptomatic leaves. Funct. Plant Biol..

[18] Magnin-Robert M., Spagnolo A., Alayi T.D., Cilindre C., Mercier L., Schaeffer-Reiss C., van Dorsselaer A., Clément C., Fontaine F. (2014). Proteomic insights into changes in wood of *Vitis vinifera* L. in response to esca proper and apoplexy. Phytopathol. Mediterr..

[19] Spagnolo A., Magnin-Robert M., Alayi T.D., Cilindre C., Mercier L., Schaeffer-Reiss C., van Dorsselaer A., Clément C., Fontaine F. (2012). Physiological changes in green stems of *Vitis vinifera* L. cv. Chardonnay in response to esca proper and apoplexy revealed by proteomic and transcriptomic analyses. J. Proteome Res..

[20] Lebon G., Wojnarowiez G., Holzapfel B., Fontaine F., Vaillant-Gaveau N., Clément C. (2008). Sugars and flowering in the grapevine (*Vitis vinifera* L.). J. Exp. Bot..

[21] Petit A.N., Baillieul F., Vaillant-Gaveau N., Jacquens L., Conreux A., Jeandet P., Clément C., Fontaine F. (2009). Low responsiveness of grapevine flowers and berries at fruit set to UV-C irradiation. J. Exp. Bot..

[22] Vaillant-Gaveau N., Maillard P., Wojnarowiez G., Gross P., Clement C., Fontaine F. (2011). Inflorescence of grapevine (*Vitis vinifera* L.): A high ability to distribute its own assimilates. J. Exp. Bot..

[23] Martínez-Lüscher J., Kizildeniz T., Vučetić V., Dai Z., Luedeling E., van Leeuwen C., Gomès E., Pascual I., Irigoyen J.J., Morales F. (2016). Sensitivity of grapevine phenology to water availability, temperature and CO_2_ concentration. Front. Environ. Sci..

[24] Paolinelli-Alfonso M., Villalobos-Escobedo J.M., Rolshausen P., Herrera-Estrella A., Galindo-Sánchez C., López-Hernández J.F., Hernandez-Martinez R. (2016). Global transcriptional analysis suggests *Lasiodiplodia theobromae* pathogenicity factors involved in modulation of grapevine defensive response. BMC Genom..

[25] Holzapfel B.P., Smith J.P. (2012). Developmental stage and climatic factors impact more on carbohydrate reserve dynamics of shiraz than cultural practice. Am. J. Enol. Vitic..

[26] Pellegrino A., Clingeleffer P., Cooley N., Walker R. (2014). Management practices impact vine carbohydrate status to a greater extent than vine productivity. Front. Plant Sci..

[27] Li C., Lu J., Xu X.F., Hu R.L., Pan Y.J. (2012). pH-switched HRP-catalyzed dimerization of resveratrol: A selective biomimetic synthesis. Green Chem..

[28] Berger S., Sinha A.K., Roitsch T. (2007). Plant physiology meets phytopathology: Plant primary metabolism and plant–pathogen interactions. J. Exp. Bot..

[29] Rojas C.M., Senthil-Kumar M., Tzin V., Mysore K.S. (2014). Regulation of primary plant metabolism during plant–pathogen interactions and its contribution to plant defense. Front. Plant Sci..

[30] Reis P., Magnin-Robert M., Nascimento T., Spagnolo A., Abou-Mansour E., Fioretti C., Clément C., Rego C., Fontaine F. (2016). Reproducing Botryosphaeria dieback foliar symptoms in a simple model system. Plant Dis..

[31] Spagnolo A., Magnin-Robert M., Alayi T.D., Cilindre C., Schaeffer-Reiss C., van Dorsselaer A., Clément C., Larignon P., Ramirez-Suero M., Chong J. (2014). Differential responses of three grapevine cultivars to Botryosphaeria dieback. Phytopathology.

[32] Dixon R.A., Achnine L., Kota P., Liu C.J., Reddy M.S.S., Wang L.J. (2002). The phenylpropanoid pathway and plant defence—A genomics perspective. Mol. Plant Pathol..

[33] Lambert C., Bisson J., Waffo-Teguo P., Papastamoulis Y., Richard T., Corio-Costet M.F., Merillon J.M., Cluzet S. (2012). Phenolics and their antifungal role in grapevine wood decay: Focus on the Botryosphaeriaceae family. J. Agric. Food Chem..

[34] Lima M.R.M., Dias A.C.P. (2012). *Phaeomoniella chlamydospora*-induced oxidative burst in *Vitis vinifera* cell suspensions: Role of NADPH oxidase and Ca^2+^. J. Phytopathol..

[35] Chang X.L., Heene E., Qiao F., Nick P. (2011). The phytoalexin resveratrol regulates the initiation of hypersensitive cell death in *Vitis* cell. PLoS ONE.

[36] Valtaud C., Foyer C.H., Fleurat-Lessard P., Bourbouloux A. (2009). Systemic effects on leaf glutathione metabolism and defence protein expression caused by esca infection in grapevines. Funct. Plant Biol..

[37] Letousey P., Baillieul F., Perrot G., Rabenoelina F., Boulay M., Vaillant-Gaveau N., Clément C., Fontaine F. (2010). Early events prior to visual symptoms in the apoplectic form of grapevine esca disease. Phytopathology.

[38] Camps C., Kappel C., Lecomte P., Leon C., Gomes E., Coutos-Thevenot P., Delrot S. (2010). A transcriptomic study of grapevine (*Vitis vinifera* cv. Cabernet-sauvignon) interaction with the vascular ascomycete fungus *Eutypa lata*. J. Exp. Bot..

[39] Ramirez-Suero M., Benard-Gellon M., Chong J., Laloue H., Stempien E., Abou-Mansour E., Fontaine F., Larignon P., Mazet-Kieffer F., Farine S. (2014). Extracellular compounds produced by fungi associated with Botryosphaeria dieback induce differential defence gene expression patterns and necrosis in *Vitis vinifera* cv. Chardonnay cells. Protoplasma.

[40] Vogel C., Marcotte E.M. (2012). Insights into the regulation of protein abundance from proteomic and transcriptomic analyses. Nat. Rev. Genet..

[41] Balasubramanian V., Vashisht D., Cletus J., Sakthivel N. (2012). Plant β-1,3-glucanases: Their biological functions and transgenic expression against phytopathogenic fungi. Biotechnol. Lett..

[42] Baggiolini M. (1952). Les stades repères dans le développement annuel de la vigne et leur utilisasion pratique. Rev. Romande d’Agric. d’Arboric..

